# Supplementary material for: Sustainably powering wearable electronics solely by biomechanical energy
Source: Nat Commun. 2016 Sep 28;7:12744. doi: 10.1038/ncomms12744 (PMC5052715; doi:10.1038/ncomms12744)
Supplement: Supplementary Figures and Supplementary Notes — Supplementary Figures 1-11 and Supplementary Notes 1-5 [file ncomms12744-s1.pdf]

Supplementary Figures

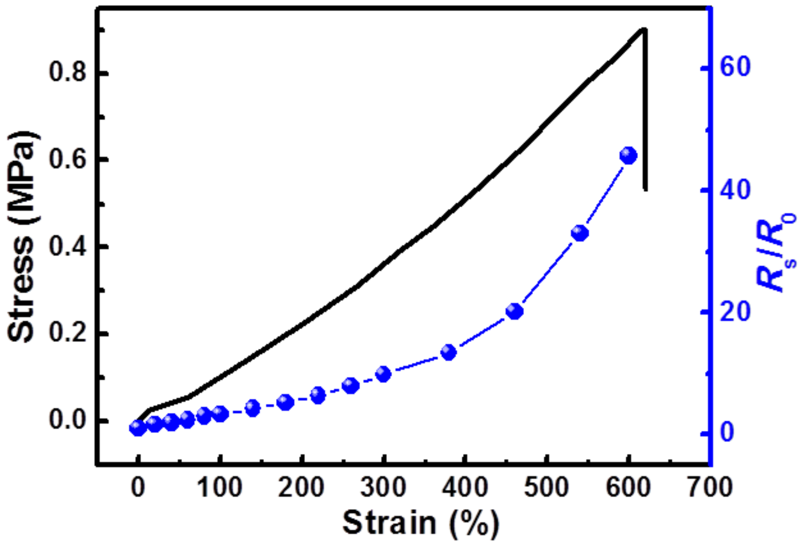

Supplementary Figure 1. Stretchability of the silicone rubber electrode. Stress-strain curve (black curve) and Relationship between resistance and tensile strain (blue line+symbol).

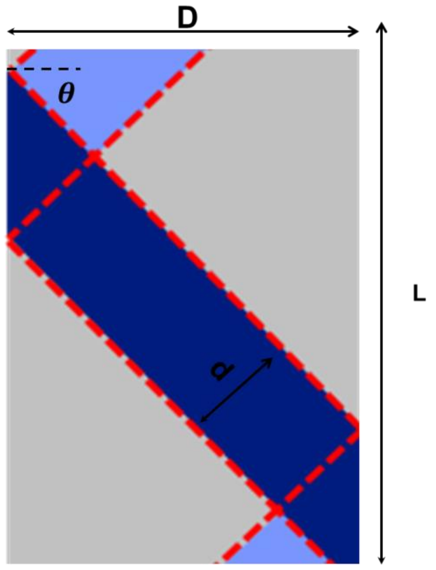

Supplementary Figure 2. Schematic diagram of the triboelectrific area of the TENG.

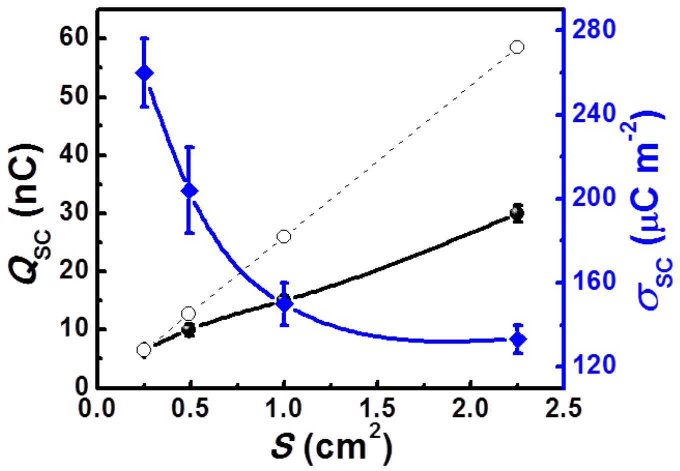

Supplementary Figure 3. Charge and charge density vs. triboelectrific area, dotted line shows the ideal charge with a constant charge density.

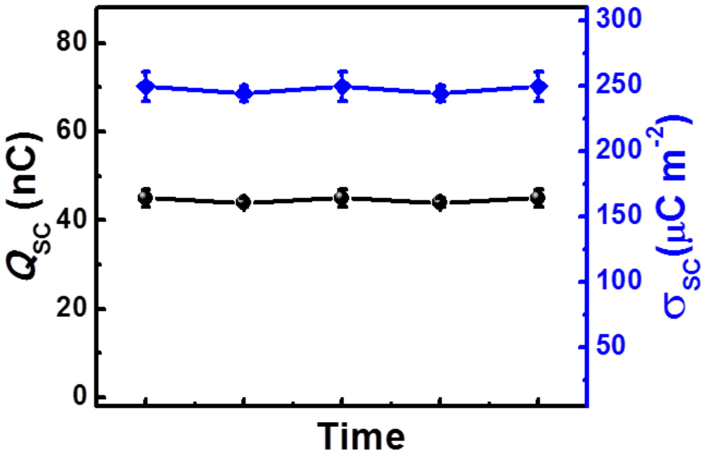

Supplementary Figure 4. Charge and charge density of the TENG-tube with a diameter of 3 mm.

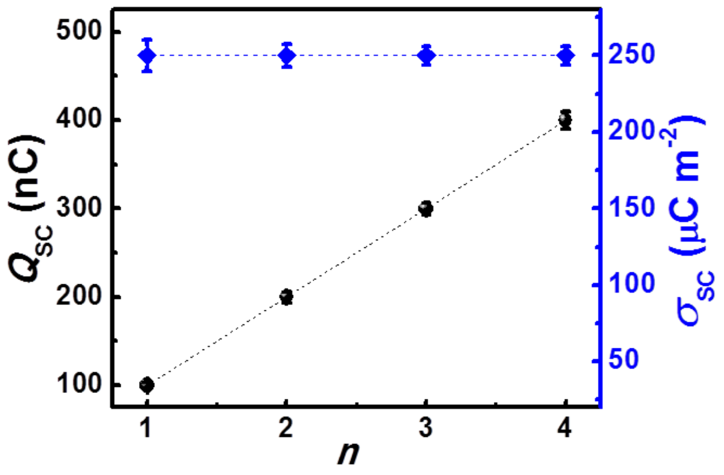

Supplementary Figure 5. Charge and charge density of the multi-TENG-tube in parallel.

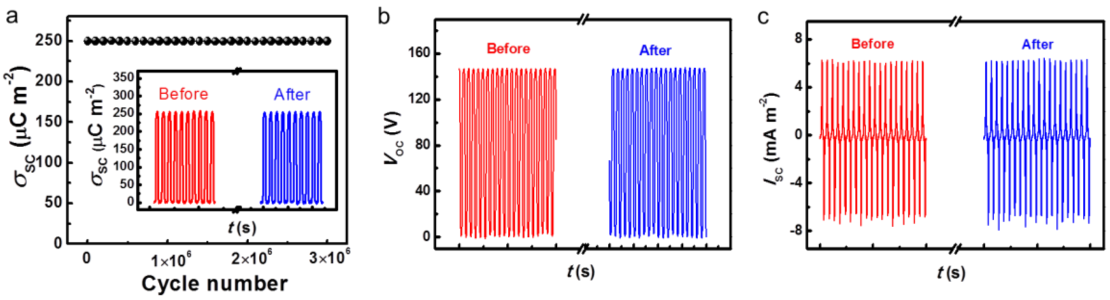

Supplementary Figure 6. Stability of the TENG, (a) charge density as function of cycle number, inset shows the charge density of the TENG before and after 3 million cycles at 10 Hz, (b) open-circuit voltage and (c) short-circuit current before and after 3 million cycles at 10 Hz.

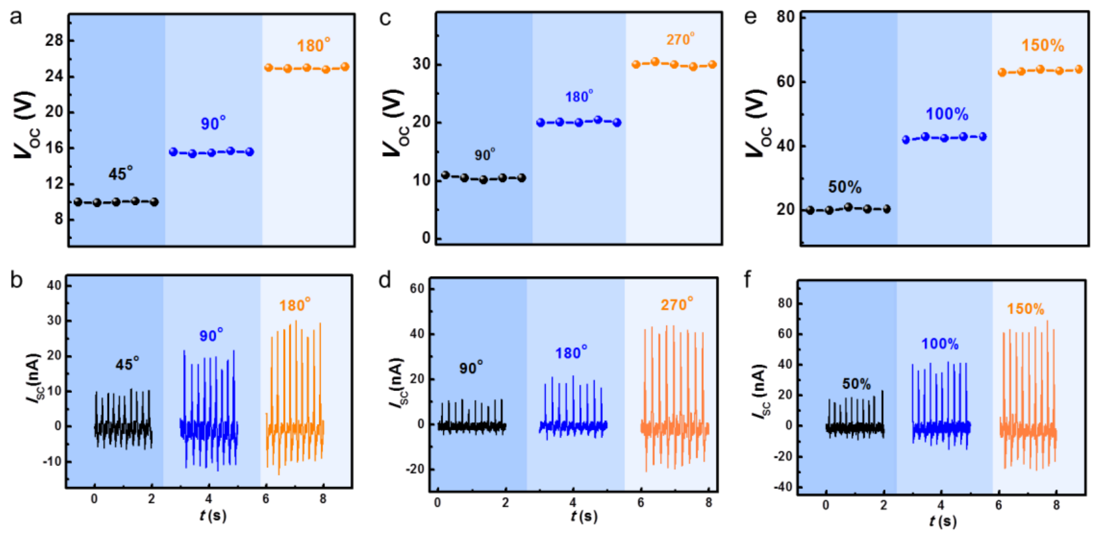

Supplementary Figure 7. Open-circuit voltage and short-circuit current of the TENG (**a, b**) under bending motion, (**c, d**) under twisting motion, (**e, f**) under lengthening motion.

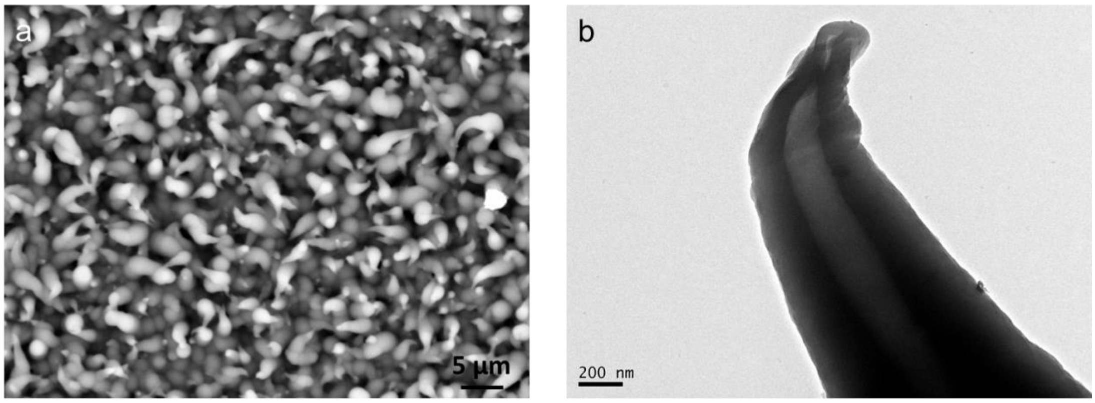

Supplementary Figure 8. SEM (a) and TEM (b) images of hPPy.

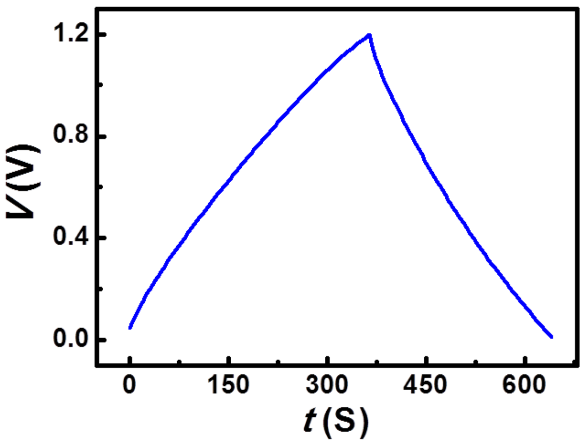

Supplementary Figure 9. Charging-discharging curve of the supercapacitor at a current density of 4 μA.

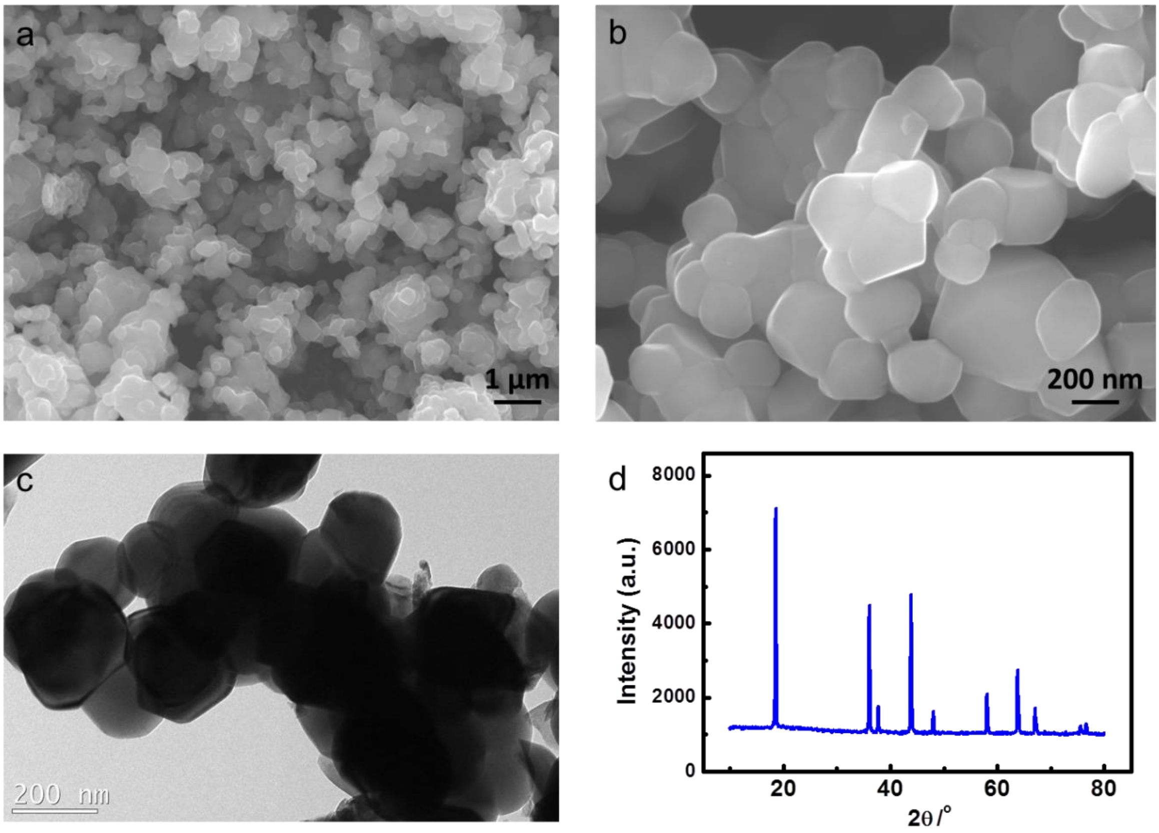

Supplementary Figure 10. SEM (a) and (b), TEM (c) images and XRD pattern (d) of $LiMn_2O_4$.

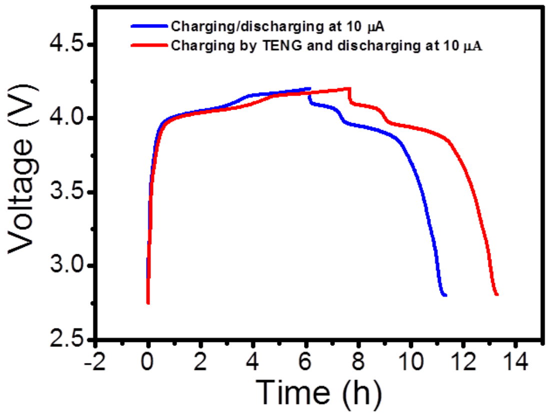

Supplementary Figure 11. Charging-discharging curve of the LIB at a current density of 10 µA (Blue line) and charging by pressing 5 TENG-tubes connected in parallel at 10 Hz with a shaker then discharing at 10 µA (Red line).

Supplementary notes

Supplementary note 1: Derivation and optimization of the effective contact area: The parameters of the tube TENG are as shown in Supplementary Fig. 2, in which *D* is the width of the TENG when it is fully pressed, *L* is the total length, θ is the angle between the contact stripe and the width *D* side, and *d* is the width of the contact stripe. There are *N* turns of contact stripe in the whole TENG, and each of them can contribute contact area of *S*. They can be deducted as:

$$N = \frac{L}{2D\tan\theta} \tag{1}$$

$$S = 2d\left(\frac{D}{\cos\theta} - \frac{d}{\sin 2\theta}\right) \tag{2}$$

Multiplying these two parameters we can deduct the total contact area as:

$$S_{\text{Total}} = N \cdot S = \frac{Ld}{\sin\theta}\left(1 - \frac{d}{2D\sin\theta}\right) \tag{3}$$

This total area is considered as a function of *d*, so that the maximum value of it can achieve.

When $d = D\sin\theta$:

$$S_{\text{Total,max}} = \frac{LD}{2} \tag{4}$$

Supplementary note 2: Effect of contact area on the charge density: We did the verifying experiment to prove the effect of contact area on the charge density. The experiments were proceeded using the basic contact-separation TENG with the areas of contacting materials ranging form 5 × 5 mm to 15 × 15 mm (5 × 5 mm, 7 × 7 mm, 10 × 10 mm, and 15 × 15 mm). For each time of measurement, we pressed with hand (with nitrile glove on) to ensure full contact. The results are demonstrated in Supplementary Fig.3, when contact area increases, the charge density of TENG drops fast while its charge is improved more slowly than linear speed (dotted line). This phenomenon can be explained by that the ineffective contact area increase with the increasing of the area, since the transfer distance of triboelectric charge is only 0.1-0.2 nm and there are no ideal smooth flat with a large area of square centimeter or larger level.

Supplementary note 3: Calculation of the equivalent galvanostatic current: Firstly, the charging capacitance (C) of the supercapacitor is calculated from galvanostatical charging curve from supplementary Fig. 9 by equation:

$$C = I \times t / V \quad (5)$$

where I is charging current, t is charging time and V represents the voltage window. From supplementary Fig. 9, the C is calculated to be 1.2 mF. Then, the equivalent galvanostatic current can be calculated by equation:

$$I = C \times V / t \quad (6)$$

From Fig. 4e, the value of I is 1.4 μA.

Supplementary note 4: Synthesis of hPPy: Pyrrole monomer (Py, Capchem, 99%) was twice distilled prior to use. The p-Toluenesulfonic acid (TOSH, China Medicine Group, AR) and sodium p-toluenesulfonic (TOSNa, China Medicine Group, CP) were used as received. The polymerization solution contained 300 mM pyrrole, 100 mM TOSH, and 400 mM TOSNa. The electrodeposition was carried out in a three-electrode cell with titanium sheet as working electrode and counter electrode, and a saturated calomel electrode (SCE) as refer electrode. The hPPy films were deposited on titanium electrodes by a pulse potentiostatic method, where the pulse parameter are a high potential of 0.75 V vs SCE, a high potential period of 0.04 s, a low potential of -0.2 V vs SCE and a low potential period of 0.12 s.

Supplementary note 5: Synthesis of $LiMn_2O_4$: The $LiMn_2O_4$ samples were prepared by solid-state reaction. All chemical reagents used in the experiments were analytical grade. Stoichiometric amounts of Li_2CO_3, Mn_3O_4 were wet ball-milled for 48 h. The milling intensity was 500 rpm and the ball-to-powder weight ratio was 5:1. After drying, the mixture of starting materials was dry ball-milled for 2 h, then precalcined at 450 °C for 6 h in the air. After slow cooling to room temperature, the calcined power was grinded by ball milling for 2 h and finally calcined at 750 °C for 12 h.
